# Genetic Determinants of Response to Statins in Cardiovascular Diseases

**DOI:** 10.2174/011573403X267793231220114042

**Published:** 2024-01-09

**Authors:** Ghazaleh Ghorbannezhad, Shima Mehrabadi, Negar Golampour-Shamkani, Amirhossein Barjasteh, Poorya Etesamizadeh, Mohammad Tayyebi, Majid Khazaei, Seyed Mahdi Hassanian, Gordon A Ferns, Amir Avan

**Affiliations:** 1 Student Research Committee, Faculty of Medicine, Mashhad University of Medical Sciences, Mashhad, Iran;; 2 Medical Genetics Research Center, Mashhad University of Medical Sciences, Mashhad, Iran;; 3 Department of Cardiology, Faculty of Medicine, Mashhad University of Medical Sciences, Mashhad, Iran;; 4 Metabolic Syndrome Research Center, Mashhad University of Medical Sciences, Mashhad, Iran;; 5 Brighton & Sussex Medical School, Division of Medical Education, Falmer, Brighton, Sussex BN1 9PH, UK

**Keywords:** Cardiovascular disease, pharmacogenetics, statin, drug-gene interactions, FDA, cardiology, Atorvastatin, fluvastatin

## Abstract

Despite extensive efforts to identify patients with cardiovascular disease (CVD) who could most benefit from the treatment approach, patients vary in their benefit from therapy and propensity for adverse drug events. Genetic variability in individual responses to drugs (pharmacogenetics) is considered an essential determinant in responding to a drug. Thus, understanding these pharmacogenomic relationships has led to a substantial focus on mechanisms of disease and drug response. In turn, understanding the genomic and molecular bases of variables that might be involved in drug response is the main step in personalized medicine. There is a growing body of data evaluating drug-gene interactions in recent years, some of which have led to FDA recommendations and detection of markers to predict drug responses (*e.g.*, genetic variant in VKORC1 and CYP2C9 genes for prediction of drug response in warfarin treatment). Also, statins are widely prescribed drugs for the prevention of CVD. Atorvastatin, fluvastatin, rosuvastatin, simvastatin, and lovastatin are the most common statins used to manage dyslipidemia. This review provides an overview of the current knowledge on the pharmacogenetics of statins, which are being used to treat cardiovascular diseases.

## 1 INTRODUCTION

The study of the response to a drug within an individual or across the population dates back circa the 1950s. Friedrich Vogel of Heidelberg, for the first time, coined the term “pharmacogenetics” in 1959, Germany, to refer to this concept. It is considered one of the essential tools of personalized medicine. Personalized medicine aims to “treat the patient, not the disease” by taking into account every individual-specific characteristics, such as age, sex, and genetics [1]. Pharmacogenetics intends to use treatments tailored to each person's specific genetic characteristics to optimize drug efficacy and reduce potential adverse drug reactions (ADR). For the past decades, there has been a growing enthusiasm to implement pharmacogenetics from bench to bedside; however, it is confined to the laboratory and specialized therapeutic areas for the most part [2]. In this context, pharmacogenetics has been applied in various medical fields, including psychology, psychiatry, antiviral, and so forth. One of the areas in which this knowledge has been highly emphasized is cardiology [3]. The last century has witnessed an exponential rise in cardiovascular diseases-related deaths, making them the leading cause of death globally by a large margin. Only in 2016, an estimated 17.9 deaths occurred as a result of CVDs, which accounted for 31% of deaths in that year [4]. Interestingly enough, genetic variations result in the development of cardiovascular diseases and make up for wide inter-individual variability in responses to CVD drugs [3, 5].

Pharmacogenetic-based drug selections and personalized diagnostic approaches might shed new light on the treatment, management, and prevention of cardiovascular diseases. Several identified polymorphisms are believed to affect the metabolism, transport, and target of commonly prescribed drugs and modify the expected treatment outcome [6].

Kindled by recent signs of progress in the concept of precision genomic medicine, the landscape of a personalized medical approach rather than a one-dose-fits-all approach may not be out of reach. The present review aimed to demonstrate how genetic polymorphisms can modulate individuals' responses to commonly prescribed statin drugs to prevent cardiovascular disorders and provide a perspective of what has been achieved regarding this topic/field thus far.

## 2 PHARMACOGENETICS IN CAD

Statins reduce CAD-associated events and act as anti-inflammatory agents by affecting lipids and lowering cholesterol levels [7, 8]. Many studies have investigated the interaction between statin group drugs and pharmacogenetics in the case of cardiovascular events. There are seven most prescribed statins (atorvastatin, lovastatin, fluvastatin, rosuvastatin, simvastatin, pitavastatin, and pravastatin), as shown in Table **1**.

## 3 ATORVASTATIN

**Fig. (1):**
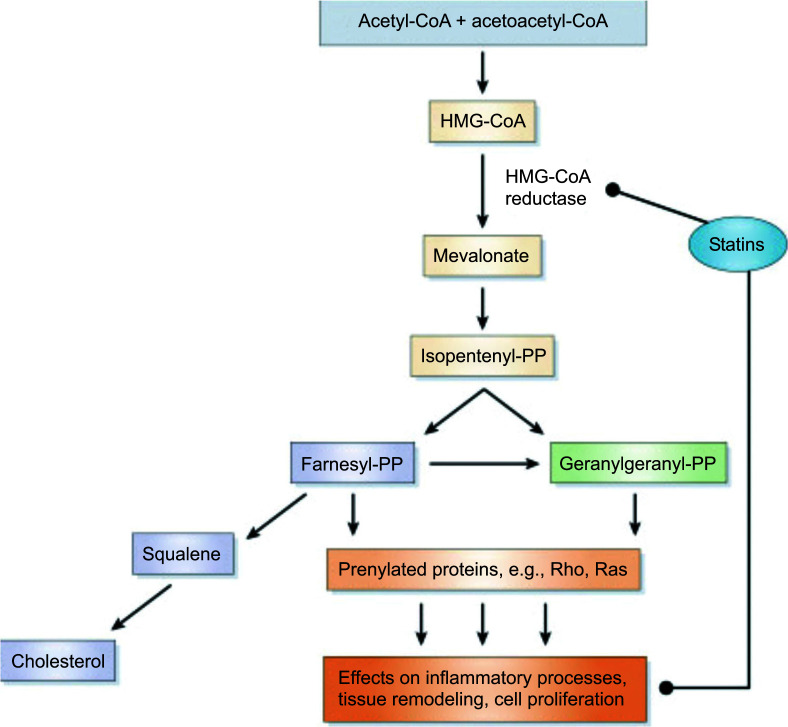
Pathway of cholesterol biosynthesis (image obtained by Cholesterol: Synthesis, Metabolism, and Regulation).

Most global guidelines on CVD treatment consider statins as the primary line of medicine for lipid-lowering effects [7]. However, statins’ lipid-lowering response from one person to another is variable. These differences have been related to genetic and environmental factors [8]. Variations in genes associated with statin and lipid digestion have been proposed as significant statin reaction elements [9]. Up to now, three GWASs have explored genetic variation and statin response [10-12]. Polymorphisms in genes, such as HMGCR, ABCB1, and CYP7A1, have shown variations in statins’ efficacy [13-15]. Liver cholesterol synthesis is limited by HMG-CoA reductase function, which is responsible for HMG-CoA to mevalonate exchange (Fig. **1**).

Statins reversibly and competitively inhibit HMG-CoA reductase, like atorvastatin. The viability and efficiency of atorvastatin have been affirmed by 200 randomized clinical trials (RCTs), with adequate clinical proof among all statins. It should pass through cytochrome P450 3A4 to construct drugs' active metabolites. It is responsible for the decreased amount of *de novo* cholesterol synthesis. Meanwhile, the expression of receptors for LDL in hepatocytes has been reported to be increased. This change results in a decreased amount of LDL-C in the blood. A dull mechanism that elevates triglycerides and, however, slightly increases levels of HDL-C exists [7].

There are multiple genes involved in atorvastatin’s metabolism and effectiveness, but the most important ones are as follows: ABCB1, a transporter of the drug; CETP, APOAI, and CYP7A1, genes involved in lipid metabolism; HMGCR, the rate-limiting enzyme involved in cholesterol metabolism; and CYP3A4, a chief enzyme involved in maintaining the drug metabolism.

An investigation (PROVE IT-TIMI 22) demonstrated a significant association between rs7412 and LDL-C level reduction. LDL-C level reduction with atorvastatin *was* more significant in carriers of apolipoprotein E ε2 versus ε4 (*p*=0.00039) [16]. This may be related to the finding, which showed a lower baseline LDL-C level in individuals homozygous for ε2 than in individuals homozygous for ε4 [17]. There are controversial studies that suggest an association between carriers of ε2 and an increased statin response or decreased cardiovascular outcomes [18, 19].

Drug transportation is often due to the activity of a P-glycoprotein encoded by the ABCB1 gene. Lack of association between ABCB1 polymorphisms (ABCB1 3435C=T and G2677T=A) and statin response in a study conducted by Poduri *et al.* has been found to be in contrast with previous studies on the relativeness of the mentioned polymorphisms and baseline LDL-C levels in Caucasians [10, 20, 21]. A polymorphism placed in the promoter region of ABCB1 (414A=G) has been shown to be involved in a more considerable increase of HDL-C following atorvastatin therapy [21].

OATP1B1 is encoded by the SLCO1B1 gene. OATP1B1 is expressed on the membrane of hepatocytes and eases the statin uptake of the liver. One variant of SLCO1B, rs4149056, modified the trafficking of the shipping polypeptide to the cell surface, bringing about raised plasma levels of atorvastatin. Enhanced risk of systemic adverse events may be a result of atorvastatin’s cholesterol-lowering ability [22]. Two polymorphisms (rs4149056 and rs2306283) have also been reported to be associated with the transporter function of OATP1B1 and altering atorvastatin pharmacokinetics [23].

CYP isoenzyme systems are responsible for all statins’ metabolism, except for pravastatin [24]. There are multiple metabolizing enzyme isoforms in the CYP system [23]. Atorvastatin is primarily metabolized by the CYP3A4 isoenzyme [25]. Elevated blood statin concentration and an increased risk of severe muscle complications are a result of inhibition of CYP3A4 [26].

Studies considering pharmacogenetics and CYP3A4 (-290A=G) are conflicting. Kajinami *et al*. reported that CYP3A4 (-290A=G) associated with atorvastatin therapy resulted in higher post-treatment LDL-C, whereas the ACCESS trial by Thompson *et al*. did not find any significant response between lipid profile and atorvastatin therapy [27, 28]. It has been reported that statin dose requirements could be influenced by the CYP3A4*2. In particular, patients with reduced activity of this SNP require 20-60% of atorvastatin's standard dose [29]. CYP2D6 has also been linked to statin metabolism, and the CYP2D6*4 allele may induce atorvastatin-caused muscle pain [30].

HMGCR is the main statin therapy target and HMGCR SNP29GG reduces LDL-C levels after atorvastatin therapy [21]. *Among other genes, ABCG8* and *CYP7A1* play significant roles in the excretion from the liver to the bile. Patients with high lipid profile treated with atorvastatin, and with *ABCG8 1199A*, exhibited reduced TG and TC, compared to *CYP7A1 A‐204*. The *ABCG8 1199A* may improve the effect of *CYP7A1 C‐204* on lower TG and TC levels. However, Kajinami *et al.* concluded that the ABCG8 H19 and CYP7A1 C-204 alleles are associated with atorvastatin response [31, 32]. The availability of multiple pathways in lipid and statin response significantly increases several gene variant combinations in response to atorvastatin therapy. Poduri *et al.* found three loci interactions, including CYP7A1, rs892871AA/APOAI, and PstIP1P1/HMGCR rs12916CT, as better in decreasing LDL-C than individual polymorphisms, illustrating that multiple gene variants can influence atorvastatin therapy [21].

### 3.1 Fluvastatin

Fluvastatin is another statin with a lowering lipid effect. One of the ways that cause inter-individual differences is statin uptake, which involves OATP transporters. These transporters uptake the many statins (except fluvastatin) from the bloodstream into the liver. Polymorphism of c.521T > C, p.Val174Ala in the SLCO1B1 gene encoding OATP1B1 reduced the ability of OATP1B1 to transport active simvastatin acid from the bloodstream into the liver, and thereby enhanced the risk of statin-induced myopathy. OATP is not a fluvastatin transporter, so its polymorphisms have a minimal effect on fluvastatin [33, 34].

Another statin transporter is P-GP (encoded by ABCB1). In 2009, Keskitalo *et al.* recruited ten healthy people in two groups, one with the ABCB1 c.1236C/C-c.2677G/G-c.3435C/C genotype and another with c.1236T/T-c.2677T/T-c.3435T/T, ingested with a single dose of fluvastatin, pravastatin, lovastatin, and rosuvastatin. They reported the ABCB1 c.1236C-c.2677G-c.3435C and c.1236T-c.2677T-c.3435T haplotypes to not be significant in the interindividual variability in the pharmacokinetics of fluvastatin, pravastatin, lovastatin, and rosuvastatin [35]. MDR1 has a vital part in the bioavailability of conventional drugs, such as digoxin, doxorubicin, and vinblastine; its substrates and MDR1 polymorphisms are responsible for inter-individual differences in drug metabolism and response [36].

Bogman *et al*. reported the modulation of MDR1 activity by various statins [23]. Berkovich *et al.* followed 20 weeks of treatment of 76 familial hyperlipidemia patients with 40 mg fluvastatin. SNP genotyping was performed to determine the association between usual SNPs and haplotypes with lipid response. They showed that both INT13 and INT17 SNPs were associated with LDL decrease, not HDL or TG. While the other SNPs did not differ considerably in HDL-c and TG or LDL levels [37]. A study conducted by Dani Bercovich *et al.* demonstrated the effect of CETP on the plasma lipid level. They showed CETP to have a vital role in HDL intravascular remodeling, which may affect HDL-C and TG levels in response to statins [37]. Further, an investigation conducted by Winkelmann *et al.* demonstrated the association between CETP haplotypes and response to statin [37, 38].

A few years later, the response of three CETP SNPs with LDL and HDL to statins was shown. The authors studied 2 SNPs in CETP, -867 and Ex14/I405V, associated with the HDL-C [37]. While pravastatin was mainly excreted as unchanged, the other statins got metabolized when they passed through the liver. Simvastatin and lovastatin were metabolized mainly by cytochrome P450 and CYP3A4 [39] and fluvastatin was primarily metabolized by CYP2C9 [39, 40], and in lesser amount by CYP3A4 and CYP2C8 [39]. CYP2C9 may influence drug metabolism and cause an inter-individual difference in fluvastatin’s efficacy and tolerance. Two main CYP2C9 polymorphisms play an essential role in fluvastatin’s adverse drug reaction (ADR) and efficacy profile, including CYP2C9*2 c.430C>T-rs1799853 and CYP2C9*3 c.1075A>C-rs1057910. These polymorphisms reduce the efficacy of cytochrome p450 and increase their bloodstream level [41]. CYP2C9*3 polymorphism reduced the efficacy of cytochrome p450 more than CYP2C9*2 [42]. For proving that, in 2013, Milosevic *et al.* recruited 52 renal transplant recipients (RTRs) and control group (52 individuals) genotyping for CYP2C9*2, *3. Finally, they demonstrated an association between fluvastatin-induced ADRs in RTRs and genetic disposition variants in the CYP2C9 [42].

CYP2D6 is another crucial enzyme in pharmacogenetics. The enzyme polymorphisms can influence its ADR or efficacy. Its important polymorphism, which affects fluvastatin’s efficacy, is CYP2D6*4 c.1846G>A-rs3892097 [41]. In 2006, Zuccaro *et al.* recruited 50 statin-treated patients with myopathy and 50 without myopathy as the control group. They showed CYP2D6 poor metabolizer (CYP2D6*4) as associated with increased statins' efficacy [43]. Statin elimination is another process that occurs in the liver. Some transporters transport the statins out of the liver after they are metabolized. ABCG2 and BRCP are two transporters that eliminate statins *via* the intestine or gall bladder. ABCG2 and BRCP polymorphisms have essential roles in fluvastatin’s ADR or efficacy [41, 42]. Its important polymorphism is c.421C>A-rs2231142, decreasing statin’s elimination rate. In 2009, a crossover study recruited five healthy individuals with *ABCG2* c.421A/A, 4 cases with c.421C/A, and 23 with c.421C/C genotype treated with fluvastatin. The plasma concentration-time curve area of fluvastatin was more significant in participants with A/A genotype than C/A or C/C genotype [44].

ApoE is a gene located on chromosome 19 and has a role in preventing disorders' effects on heart and blood vessels. ApoE ɛ2(c.334T-c.472T) is one of the ApoE polymorphisms that can influence fluvastatin’s efficacy [41]. In 2005, Thompson *et al.* recruited 2735 individuals on statins, with half of the sample on atorvastatin, while the other half of the sample was on other statins (including fluvastatin, lovastatin, pravastatin, and simvastatin). There were 477 on fluvastatin. They have genotyped 43 SNPs in 16 genes. They discovered ApoE ɛ2(c.334T-c.472T) to be a rare SNP, but having a significant effect on statin therapy and lowering of LDL [28].

HMG CoA is an enzyme cholesterol-producing pathway, converting the HMG CoA to mevalonate for the production of cholesterol. Its important polymorphism is c.2457 + 117T>G-rs17238540 [8]. From 1993 to 2006, Donnelly *et al.* recruited 1601 persons. All patients were genotyped for rs17238540. They concluded that individuals heterozygous for the G allele of rs17238540 in the HMGCoAR gene might respond less than homozygous to statins, such as fluvastatin therapy, in terms of total cholesterol and lowering triglycerides [45].

### 3.2 Rosuvastatin

Rosuvastatin, a thoroughly synthetic member of the statins family, has been shown to have remarkable efficacy in enhancing the lipid profile compared to other molecules of the same category [46]. Like all other statins, rosuvastatin plays its role in reducing cholesterol's endogenous synthesis by inhibiting HMG-CoA reductase [46, 47].

It has been pinpointed that polymorphism in genes associated with rosuvastatin metabolism or the ones connected with proteins involved in rosuvastatin transport or hepatic uptake can account for the inter-subject variability of drug responses. In light of this fact, different studies have been carried out to discover the variants relevant to a more significant reduction in LDL levels in cases taking rosuvastatin. ABC G2, a member of the ABC family, is responsible for the cellular efflux of xenobiotics. Rosuvastatin is also one of its substrates. Some studies' results support the fact that specific polymorphisms can lead to higher efficacy of the drug in reducing LDL levels; for instance, individuals with the c.421AA genotype showed a more significant decrease in the LDL levels compared to the ones with the c.421CC genotype [35, 48].

The same effect was observed amongst subjects with rs2199936 A polymorphism [49]. In 2014, Ferrari *et al.* found that three polymorphisms, rs1045642, rs1128503, and rs1128503, resulted in a diminished LDL-lowering potency of rosuvastatin [50]. Polymorphism in another statins’ transporter gene, SLCO1B1, which is considered to have an essential role in statins’ disposition, has also been shown to influence the response to rosuvastatin; as an example, higher rosuvastatin level has been detected in subjects with rs4149056 (c.521T>C) polymorphism. [51]. However, several studies have not been able to discover an apparent relationship between this polymorphism and the action of rosuvastatin [52, 53]. APEO, a protein involved in fat metabolism, is known to be associated with cardiovascular diseases.

Interestingly, rosuvastatin’s efficiency has been reported to be positively affected by *APOE* rs4420638 G allele polymorphism, whereas the response to the therapy would debilitate in rs71352238 C allele carriers [49, 54, 55]. Still, so many other genes’ polymorphisms, such as in LPL, PCSK9, SOD2, KIF6, *etc*., are known to be determinants of inter-individual variability in response to rosuvastatin [41, 49, 54-59].

### 3.3 Simvastatin

Simvastatin is one of the critical prescribed drugs in hypercholesterolemia and, consequently, atherosclerotic diseases. It reduces the biosynthesis of the low-density lipoprotein cholesterol in hepatocytes by inhibiting the HMG-CoA reductase enzyme. Simvastatin's effect on individuals differs from each other, and this belongs to the particular genotypes that interact with drug functions [60]. SLCO1B1 and CYP3A4/5 are some instances. Expression of CYP3A4 is genuinely affected by intron 6 SNP rs35599367 in the CYP3A4*22 gene, which significantly reduces CYP3A4 mRNA expression enzyme activity [29]. Decreasing activation of CYP3A4 is associated with lower drug metabolism and high simvastatin levels in the bloodstream. These enable a better function of the drug. A prospective population-based cohort study evaluated the association of the CYP3A4 intron 6 T-variant alleles with increased TC and LDL reduction in response to simvastatin therapy [29].

### 3.4 Lovastatin

Lovastatin, which is also known by the brand name Mevacor, is one of the most common drugs used for hypercholesteremia. Many studies have not focused on the effect of genetic polymorphisms on the efficacy of lovastatin; however, cytochrome P450 CYP3A is an essential tool in the metabolism and elimination of lovastatin. CYP3A4 and CYP3A5 oxidized lovastatin into various metabolites in the intestinal wall and liver [40] However, the polymorphism of CYP3A5 also contributes to the biotransformation of lovastatin [61-63]. CYP3A5 and CYP3A5*3 granted low or undetectable CYP3A5 expression due to the mutation of 6986A.G [64-69].

An investigation evaluated LDL response in patients taking lovastatin, simvastatin, or atorvastatin. The subjects with CYP3A5*3/*3 polymorphism showed better response than *1 carriers [64]. However, two other studies focused on the APO variants and how they affect the lipid-lowering qualities of lovastatin, and their results have been found to be contradictory. In contrast, one study showed that TC and LDL lowering had significantly downgraded in a male participant with the APOE ε4 allele. Another study suggested that ε2, ε3, or ε4 variant alleles did not influence lovastatin’s efficacy whatsoever [65].

### 3.5 Pravastatin

*Pravastatin* is a lipoprotein-lowering drug that acts mainly by inhibiting HMG-CoA reductase's function by occupying the enzyme’s active site. OATP1B1 is a membrane influx transporter that regulates the transport of pravastatin into the hepatic cells [66]. Researches indicate that variation in the SLCO1B1 gene is associated with response to pravastatin. In a study involving the Chinese population, a variant SLCO1B1 rs4149056 C allele was shown to be associated with an 8% lower reaction to the drug in question (*p*=0.03) [67]. Another study comprising Japanese patients had provided a similar result, only with a 6% lower response rate (*p*<0.05) [68]. Moreover, a study in 2006 with heterozygous carriers of the SLCO1B1*15 allele who carried both the rs4149056 C allele and the rs2306283 G allele also showed a lower reduction in TC and LDL levels [69].

This drug's effect on lowering cholesterol is also attributed to inhibiting the synthesis of very low-density lipoproteins, which are the precursor to LDL. Thus, polymorphism in genes involved in different levels of cholesterol synthesis may affect pravastatin's efficacy. In line with this, patients with HMGCR haplotype 7 and rs17244841 and rs17238540 polymorphisms have been reported to show a reduction in response to pravastatin [70, 71]. Paraoxonase (PON) is an enzyme carried by HDL-C. A study in 2001 attempted to examine the effect of the PON genotype on HDL-C and apolipoprotein A-I (apo AI) in response to pravastatin treatment in male subjects and found Q192R polymorphism as associated with better HDL and response to pravastatin [72]. Other studies have suggested that apolipoprotein (Apo) E and Apo A-IV isoform variation (-75G>A) may result in a smaller HDL increase with pravastatin (*p*=0.008) [73]. While some studies have stated that specific apolipoprotein E alleles (ε2,ε3,ε4) might be associated with increased efficacy of pravastatin [69, 74]. Also, patients with the APOEε2 (*2) allele showed an increased reduction in TC and LDL cholesterol levels in comparison to those with no carriers [74]. In another study on the effect of pravastatin on the progression of CAD, the Taq1B B1 allele (rs708272) of the CETP gene was shown to be associated with a reduction in the progression of coronary atherosclerosis [75].

The -514C/T polymorphism of the hepatic lipase gene was also associated with a significant HDL increase [76]. The TLR4 Asp299Gly polymorphism (rs4986790) was associated with a decreased risk of cardiovascular events in response to pravastatin therapy [77]. Furthermore, carriers of the rs20455 variation KIF6, a gene encoding the kinesin family member with six proteins, were associated with a decreasing risk of myocardial infarction recurrence in response to pravastatin therapy [78]. Simultaneously, carriers of the Taq1BB2 allele of the CETP gene were reported to be at increased risk of CVD-related death under some circumstances [79].

## CONCLUSION

Pharmacogenetics is proliferating nowadays. It is currently known as one of the many fields in pharmacology. It exhibits an association between pharmacogenetics and diseases with unique genetic patterns in response to different drugs for preventing threatening disorders. Statins are widely used to cure or prevent cardiovascular disorders. Understanding the genetic variations and pharmacogenetics relations can increase statins' efficacy in preventing cardiovascular disorders. In other words, pharmacogenomics data provide crucial insights into the mechanisms of disease and response to drugs. A summary of the most important genes and their polymorphisms along with an overview of the efficacy of drugs in pharmacodynamics are provided in Table **2**.

## Figures and Tables

**Table 1:** The most commonly used statins and the genes that may affect their efficacy.

Drug	Gene
**Atorvastatin**	HMGCR, ABCB1, CYP7A1, APO, CYP3A4, CYP2D6, *ABCG8*
**Fluvastatin**	CYP2D6, CYP2C9, ABCG2/BRCP, ApoE, HMGCoR, MDR1, CETP, SLCO1B1, ABCB1
**Rosuvastatin**	-
**Simvastatin**	SLCOB1, CYP3A4
**Lovastatin**	APOA5, APOE, CYP3A5, CYP3A4
**Pravastatin**	SLCO1B1, HMGCR, CETP
**Pitavastatin**	SLCOB1, CYP2C9, CYP2C8 (minimally)

**Table 2:** The most important genes and their polymorphisms with an overview of their efficacy of drugs in pharmacodynamic.

Gene	Polymorphism/ SNP	Drug	Patient	Efficacy (Yes or No)	References
**CYP3A4** **ABCB1** **SLCO1B1**	290A=G G2677T=A RS2306283	Atorvastatin	1380 340 5745	Yes Yes YES	[1, 2]
**CYP2D6** **CYP2C9** **ABCG2/BRCP** **ApoE** **HMG-CoR** **MDR1** **CETP** **SLCO1B1** **ABCB1**	RS3892097 RS1799853/RS2740574 RS2231142 c.334T – c.472T RS17238540 INT13/ INT17 -867 Ex13/G412G 512T>C c.1236C-c.2677G-c.3435C / c.1236T-c.2677T-c.3435T	Fluvastatin	100 104 32 477 1601 76 76 32 20	YES YES YES YES YES YES YES NO NO	[3-6]
**ABCG2** **SLCO1B1** **APEO** **LPL** **PCSK9** **SOD2** **KIF6**	RS2231142 RS4149056 RS4420638/RS71352238 RS328 RS17111584 RS4880 c.2155T>C	Rosuvastatin	305 291 372/3523 378 3523 122 344	YES YES YES YES YES YES YES	[7] [8] [9, 10] [9] [10] [11] [12]
** CYP3A4**	-	Simvastatin	80	Yes	[13]
** APOA5** ** APoE** **CYP3A5**	Rs662799 Rs429358-rs7412 Rs776746	Lovastatin	657 98 46	Yes Yes Yes	[14]
**SLCOB1** **APo**E **ABCB1** **APOC1** **CETP** **HMGCR**	RS4149056 RS7412 RS1922242 RS4420638 RS708272 RS17244841/RS17238540	Pravastatin	45/66 97 1883 1976 807/812 1536	YES YES YES YES YES/YES YES	[15, 16] [16] [17] [18] [19, 20] [21]

## LIST OF ABBREVIATIONS

ADR: Adverse Drug Reactions
Apo: Apolipoprotein
apo AI: Apolipoprotein A-I
CVD: Cardiovascular Disease
CYP: Cytochrome P450
RCTs: Randomized Clinical Trials

## References

[1] Lu Y.F., Goldstein D.B., Angrist M., Cavalleri G. (2014). Personalized medicine and human genetic diversity.. Cold Spring Harb. Perspect. Med..

[2] Dias M.M., Sorich M.J., Rowland A., Wiese M.D., McKinnon R.A. (2017). The routine clinical use of pharmacogenetic tests: What it will require?. Pharm. Res..

[3] Dávila-Fajardo CL, Díaz-Villamarín X, Antúnez-Rodríguez A (2019). Pharmacogenetics in the treatment of cardiovascular diseases and its current progress regarding implementation in the clinical routine.. Genes.

[4] Wang H., Naghavi M., Allen C. (2016). Global, regional, and national life expectancy, all-cause mortality, and cause-specific mortality for 249 causes of death, 1980–2015: a systematic analysis for the Global Burden of Disease Study 2015.. Lancet.

[5] Lamoureux F., Duflot T. (2017). Pharmacogenetics in cardiovascular diseases: State of the art and implementation-recommendations of the french national network of pharmacogenetics (RNPGx).. Therapie.

[6] Zaiou M., El Amri H. (2017). Cardiovascular pharmacogenetics: A promise for genomically‐guided therapy and personalized medicine.. Clin. Genet..

[7] Ye Y.C., Zhao X.L., Zhang S.Y. (2015). Use of atorvastatin in lipid disorders and cardiovascular disease in Chinese patients.. Chin. Med. J..

[8] Mangravite L.M., Thorn C.F., Krauss R.M. (2006). Clinical implications of pharmacogenomics of statin treatment.. Pharmacogenomics J..

[9] Hutz MH, Fiegenbaum M (2008). Impact of genetic polymorphisms on theefficacy of HMG-CoA reductase inhibitors.. American journal of cardiovascular drugs : drugs, devices, and other interventions.

[10] Barber M.J., Mangravite L.M., Hyde C.L. (2010). Genome-wide association of lipid-lowering response to statins in combined study populations.. PLoS One.

[11] Link E., Parish S., Armitage J. (2008). SLCO1B1 variants and statin-induced myopathy--a genomewide study.. N. Engl. J. Med..

[12] Thompson J.F., Hyde C.L., Wood L.S. (2009). Comprehensive whole-genome and candidate gene analysis for response to statin therapy in the Treating to New Targets (TNT) cohort.. Circ. Cardiovasc. Genet..

[13] Mohassel P., Mammen A.L. (2013). Statin‐associated autoimmune myopathy and anti‐HMGCR autoantibodies.. Muscle Nerve.

[14] Su J., Xu H., Yang J. (2015). ABCB1 C3435T polymorphism and the lipid-lowering response in hypercholesterolemic patients on statins: A meta-analysis.. Lipids Health Dis..

[15] Lim M.Y.C., Tee J.R., Yau W.P., Ho H.K. (2023). A meta-analysis of the pooled impact of CYP7A1 single nucleotide polymorphisms on serum lipid responses to statins.. Front. Genet..

[16] Mega J.L., Morrow D.A., Brown A., Cannon C.P., Sabatine M.S. (2009). Identification of genetic variants associated with response to statin therapy.. Arterioscler. Thromb. Vasc. Biol..

[17] Bennet A.M., Di Angelantonio E., Ye Z. (2007). Association of apolipoprotein E genotypes with lipid levels and coronary risk.. JAMA.

[18] Nieminen T., Kähönen M., Viiri L.E., Grönroos P., Lehtimäki T. (2008). Pharmacogenetics of apolipoprotein E gene during lipid-lowering therapy: Lipid levels and prevention of coronary heart disease.. Pharmacogenomics.

[19] Zintzaras E., Kitsios G.D., Triposkiadis F., Lau J., Raman G. (2009). APOE gene polymorphisms and response to statin therapy.. Pharmacogenomics J..

[20] Kajinami K., Brousseau M.E., Ordovas J.M., Schaefer E.J. (2004). Polymorphisms in the multidrug resistance-1 (MDR1) gene influence the response to atorvastatin treatment in a gender-specific manner.. Am. J. Cardiol..

[21] Poduri A., Khullar M., Bahl A., Sehrawat B.S., Sharma Y., Talwar K.K. (2010). Common variants of HMGCR, CETP, APOAI, ABCB1, CYP3A4, and CYP7A1 genes as predictors of lipid-lowering response to atorvastatin therapy.. DNA Cell Biol..

[22] Hadjiphilippou S., Ray K.K. (2019). Cholesterol-lowering agents.. Circ. Res..

[23] Hirota T., Fujita Y., Ieiri I. (2020). An updated review of pharmacokinetic drug interactions and pharmacogenetics of statins.. Expert Opin. Drug Metab. Toxicol..

[24] Hirota T., Ieiri I. (2015). Drug–drug interactions that interfere with statin metabolism.. Expert Opin. Drug Metab. Toxicol..

[25] Bellosta S, Paoletti R, Corsini A. (2004). Safety of Statins.. Circulation.

[26] Fallah A., Deep M., Smallwood D., Hughes P. (2013). Life-threatening rhabdomyolysis following the interaction of two commonly prescribed medications.. Australas. Med. J..

[27] Kajinami K., Brousseau M.E., Ordovas J.M., Schaefer E.J. (2004). CYP3A4 genotypes and plasma lipoprotein levels before and after treatment with atorvastatin in primary hypercholesterolemia.. Am. J. Cardiol..

[28] Thompson J.F., Man M., Johnson K.J. (2005). An association study of 43 SNPs in 16 candidate genes with atorvastatin response.. Pharmacogenomics J..

[29] Wang D., Guo Y., Wrighton S.A., Cooke G.E., Sadee W. (2011). Intronic polymorphism in CYP3A4 affects hepatic expression and response to statin drugs.. Pharmacogenomics J..

[30] Frudakis T.N., Thomas M.J., Ginjupalli S.N., Handelin B., Gabriel R., Gomez H.J. (2007). CYP2D6*4 polymorphism is associated with statin-induced muscle effects.. Pharmacogenet. Genomics.

[31] Kajinami K., Brousseau M.E., Ordovas J.M., Schaefer E.J. (2004). Interactions between common genetic polymorphisms in ABCG5/G8 and CYP7A1 on LDL cholesterol-lowering response to atorvastatin.. Atherosclerosis.

[32] Wei K.K., Zhang L.R., Zhang Y., Hu X.J. (2011). Interactions between CYP7A1 A-204C and ABCG8 C1199A polymorphisms on lipid lowering with atorvastatin.. J. Clin. Pharm. Ther..

[33] Kalliokoski A., Niemi M. (2009). Impact of OATP transporters on pharmacokinetics.. Br. J. Pharmacol..

[34] Pasanen M.K., Miettinen T.A., Gylling H., Neuvonen P.J., Niemi M. (2008). Polymorphism of the hepatic influx transporter organic anion transporting polypeptide 1B1 is associated with increased cholesterol synthesis rate.. Pharmacogenet. Genomics.

[35] Keskitalo J.E., Kurkinen K.J., Neuvonen M., Backman J.T., Neuvonen P.J., Niemi M. (2009). No significant effect of ABCB1 haplotypes on the pharmacokinetics of fluvastatin, pravastatin, lovastatin, and rosuvastatin.. Br. J. Clin. Pharmacol..

[36] Hougaard Christensen M.M., Bruun Haastrup M., Øhlenschlæger T. (2020). Interaction potential between clarithromycin and individual statins—A systematic review.. Basic Clin. Pharmacol. Toxicol..

[37] Bercovich D., Friedlander Y., Korem S. (2006). The association of common SNPs and haplotypes in the CETP and MDR1 genes with lipids response to fluvastatin in familial hypercholesterolemia.. Atherosclerosis.

[38] Winkelmann B.R., Hoffmann M.M., Nauck M. (2003). Haplotypes of the cholesteryl ester transfer protein gene predict lipid-modifying response to statin therapy.. Pharmacogenomics J..

[39] Mukai Y., Narita M., Akiyama E. (2017). Co-administration of fluvastatin and CYP3A4 and CYP2C8 inhibitors may increase the exposure to fluvastatin in carriers of CYP2C9 genetic variants.. Biol. Pharm. Bull..

[40] Neuvonen P.J., Backman J.T., Niemi M. (2008). Pharmacokinetic comparison of the potential over-the-counter statins simvastatin, lovastatin, fluvastatin and pravastatin.. Clin. Pharmacokinet..

[41] Arrigoni E., Del Re M., Fidilio L., Fogli S., Danesi R., Di Paolo A. (2017). Pharmacogenetic foundations of therapeutic efficacy and adverse events of statins.. Int. J. Mol. Sci..

[42] Miroševic Skvrce N., Božina N., Zibar L., Barišic I., Pejnovic L., Macolic Šarinic V. (2013). CYP2C9 and ABCG2 polymorphisms as risk factors for developing adverse drug reactions in renal transplant patients taking fluvastatin: A case–control study.. Pharmacogenomics.

[43] Zuccaro P., Mombelli G., Calabresi L., Baldassarre D., Palmi I., Sirtori C.R. (2007). Tolerability of statins is not linked to CYP450 polymorphisms, but reduced CYP2D6 metabolism improves cholesteraemic response to simvastatin and fluvastatin.. Pharmacol. Res..

[44] Keskitalo J.E., Pasanen M.K., Neuvonen P.J., Niemi M. (2009). Different effects of the ABCG2 c.421C>A SNP on the pharmacokinetics of fluvastatin, pravastatin and simvastatin.. Pharmacogenomics.

[45] Donnelly L.A., Doney A.S.F., Dannfald J. (2008). A paucimorphic variant in the HMG-CoA reductase gene is associated with lipid-lowering response to statin treatment in diabetes: A GoDARTS study.. Pharmacogenet. Genomics.

[46] Cortese F., Gesualdo M., Cortese A. (2016). Rosuvastatin: Beyond the cholesterol-lowering effect.. Pharmacol. Res..

[47] Taheri R., Razmjou A., Szekely G., Hou J., Ghezelbash G.R. (2016). Biodesalination —On harnessing the potential of nature’s desalination processes.. Bioinspir. Biomim..

[48] Tomlinson B., Hu M., Lee V.W.Y. (2010). ABCG2 polymorphism is associated with the low-density lipoprotein cholesterol response to rosuvastatin.. Clin. Pharmacol. Ther..

[49] Chasman D.I., Giulianini F., MacFadyen J., Barratt B.J., Nyberg F., Ridker P.M. (2012). Genetic determinants of statin-induced low-density lipoprotein cholesterol reduction: the justification for the use of statins in prevention: an intervention trial evaluating rosuvastatin (JUPITER) trial.. Circ. Cardiovasc. Genet..

[50] Ferrari M., Guasti L., Maresca A. (2014). Association between statin-induced creatine kinase elevation and genetic polymorphisms in SLCO1B1, ABCB1 and ABCG2.. Eur. J. Clin. Pharmacol..

[51] Lee H.K., Hu M., Lui S.S.H., Ho C.S., Wong C.K., Tomlinson B. (2013). Effects of polymorphisms in ABCG2, SLCO1B1, SLC10A1 and CYP2C9/19 on plasma concentrations of rosuvastatin and lipid response in Chinese patients.. Pharmacogenomics.

[52] Rose R.H., Neuhoff S., Abduljalil K., Chetty M., Rostami-Hodjegan A., Jamei M. (2014). Application of a physiologically based pharmacokinetic model to predict OATP1B1 ‐related variability in pharmacodynamics of rosuvastatin.. CPT Pharmacometrics Syst. Pharmacol..

[53] Kim T.E., Shin D., Gu N. (2017). The effect of genetic polymorphisms in SLCO2B1 on the lipid‐lowering efficacy of rosuvastatin in healthy adults with elevated low‐density lipoprotein.. Basic Clin. Pharmacol. Toxicol..

[54] Hu M., Lui S.S.H., Mak V.W.L. (2010). Pharmacogenetic analysis of lipid responses to rosuvastatin in Chinese patients.. Pharmacogenet. Genomics.

[55] Zhou Q., Ruan Z.R., Yuan H., Xu D.H., Zeng S. (2013). ABCB1 gene polymorphisms, ABCB1 haplotypes and ABCG2 c.421c > A are determinants of inter-subject variability in rosuvastatin pharmacokinetics.. Pharmazie.

[56] Duarte T., da Cruz I.B.M., Barbisan F., Capelleto D., Moresco R.N., Duarte M.M.M.F. (2016). The effects of rosuvastatin on lipid-lowering, inflammatory, antioxidant and fibrinolytics blood biomarkers are influenced by Val16Ala superoxide dismutase manganese-dependent gene polymorphism.. Pharmacogenomics J..

[57] Shek A., Alieva R., Kurbanov R. (2018). Burden of familial heterozygous hypercholesterolemia in Uzbekistan: Time is muscle.. Atherosclerosis.

[58] Peters B.J.M., Pett H., Klungel O.H. (2011). Genetic variability within the cholesterol lowering pathway and the effectiveness of statins in reducing the risk of MI.. Atherosclerosis.

[59] Ruiz-Iruela C., Padró-Miquel A., Pintó-Sala X. (2018). KIF6 gene as a pharmacogenetic marker for lipid-lowering effect in statin treatment.. PLoS One.

[60] Westlind-Johnsson A., Malmebo S., Johansson A. (2003). Comparative analysis of CYP3A expression in human liver suggests only a minor role for CYP3A5 in drug metabolism.. Drug Metab. Dispos..

[61] Jacobsen W., Kirchner G., Hallensleben K. (1999). Comparison of cytochrome P-450-dependent metabolism and drug interactions of the 3-hydroxy-3-methylglutaryl-CoA reductase inhibitors lovastatin and pravastatin in the liver.. Drug Metab. Dispos..

[62] Prueksaritanont T., Gorham L.M., Ma B. (1997). *In vitro* metabolism of simvastatin in humans SBT identification of metabolizing enzymes and effect of the drug on hepatic P450s.. Drug Metab. Dispos..

[63] Jacobsen W., Kuhn B., Soldner A. (2000). Lactonization is the critical first step in the disposition of the 3-hydroxy-3-methylglutaryl-CoA reductase inhibitor atorvastatin.. Drug Metab. Dispos..

[64] Kivistö K.T., Niemi M., Schaeffeler E. (2004). Lipid-lowering response to statins is affected by CYP3A5 polymorphism.. Pharmacogenetics.

[65] Xiong Z., Cao X., Wen Q. (2019). An overview of the bioactivity of monacolin K / lovastatin.. Food Chem. Toxicol..

[66] De Angelis G. (2004). The influence of statin characteristics on their safety and tolerability.. Int. J. Clin. Pract..

[67] Zhang W., Chen B.L., Ozdemir V. (2007). SLCO1B1 521T→C functional genetic polymorphism and lipid‐lowering efficacy of multiple‐dose pravastatin in Chinese coronary heart disease patients.. Br. J. Clin. Pharmacol..

[68] Tachibana-Iimori R., Tabara Y., Kusuhara H. (2004). Effect of genetic polymorphism of OATP-C (SLCO1B1) on lipid-lowering response to HMG-CoA reductase inhibitors.. Drug Metab. Pharmacokinet..

[69] Takane H., Miyata M., Burioka N. (2006). Pharmacogenetic determinants of variability in lipid-lowering response to pravastatin therapy.. J. Hum. Genet..

[70] Jukema J.W., van Boven A.J., Groenemeijer B. (1996). The Asp9 Asn mutation in the lipoprotein lipase gene is associated with increased progression of coronary atherosclerosis. REGRESS Study Group, Interuniversity Cardiology Institute, Utrecht, The Netherlands. Regression Growth Evaluation Statin Study.. Circulation.

[71] Chasman D.I., Posada D., Subrahmanyan L., Cook N.R., Stanton V.P., Ridker P.M. (2004). Pharmacogenetic study of statin therapy and cholesterol reduction.. JAMA.

[72] Malin R., Laaksonen R., Knuuti J. (2001). Paraoxonase genotype modifies the effect of pravastatin on high-density lipoprotein cholesterol.. Pharmacogenetics.

[73] Huebbe P., Rimbach G. (2017). Evolution of human apolipoprotein E (APOE) isoforms: Gene structure, protein function and interaction with dietary factors.. Ageing Res. Rev..

[74] Peña R., Lahoz C., Mostaza J.M. (2002). Effect of apoE genotype on the hypolipidaemic response to pravastatin in an outpatient setting.. J. Intern. Med..

[75] Kuivenhoven J.A., Jukema J.W., Zwinderman A.H. (1998). The role of a common variant of the cholesteryl ester transfer protein gene in the progression of coronary atherosclerosis.. N. Engl. J. Med..

[76] Lahoz C., Peña R., Mostaza J.M. (2005). The − 514C/T polymorphism of the hepatic lipase gene significantly modulates the HDL-cholesterol response to statin treatment.. Atherosclerosis.

[77] Boekholdt S.M., Agema W.R.P., Peters R.J.G. (2003). Variants of toll-like receptor 4 modify the efficacy of statin therapy and the risk of cardiovascular events.. Circulation.

[78] Iakoubova O.A., Tong C.H., Rowland C.M. (2008). Association of the Trp719Arg polymorphism in kinesin-like protein 6 with myocardial infarction and coronary heart disease in 2 prospective trials: the CARE and WOSCOPS trials.. J. Am. Coll. Cardiol..

[79] Regieli J.J., Jukema J.W., Grobbee D.E. (2008). CETP genotype predicts increased mortality in statin-treated men with proven cardiovascular disease: an adverse pharmacogenetic interaction.. Eur. Heart J..

